# A woman with worsening abdominal pain and eosinophilia: disseminated echinococcus disease

**DOI:** 10.11604/pamj.2022.43.17.32718

**Published:** 2022-09-09

**Authors:** Petros Ioannou, Vasiliki Mavrikaki

**Affiliations:** 1 Department of Internal Medicine, University Hospital of Heraklion, Crete, Greece

**Keywords:** Echinococcus, flatworms, parasitosis, platyhelminthes

## Image in medicine

A 29-year-old woman presented to the hospital due to worsening pain in the right lower quadrant of the abdomen for 6 months. There was no association between pain and eating. No history of nausea, diarrhea, vomiting, hematochezia, melena, constipation or fever was noted. She had a soft abdomen with a deep tenderness in the right lower quadrant. Blood examination was normal except for an eosinophil count of 2,000/µL. A magnetic resonance imaging of the abdomen was performed. The presence of cysts inside the cysts aided towards the diagnosis of echinococcal disease. As seen in the figure, the enlarged liver contains many cysts that contain multiple cysts. Furthermore, serology for Echinococcus was positive. Echinococcosis (hydatid disease) is a zoonotic caused by the larvae *Echinococcus multilocularis*. Infection follows the accidental ingestion of parasite's eggs. Eggs released oncospheres that migrate to the internal organs, more commonly the liver and the lungs, where larval forms develop and form structures composed of small cysts that contain numerous hydatid cysts. Echinococcus disease is most commonly diagnosed in the Middle East, China, India, Siberia, and Alaska, while, in Europe, it is rare. The clinical presentation depends on the anatomical location of the cysts and their size. In order to reduce the likelihood of relapse, albendazole can be used preoperatively for at least one month, and can also be used in cases where a conservative method is chosen due to high-risk surgical procedure, as the perioperative mortality varies and can be as high as 4%.

**Figure 1:**
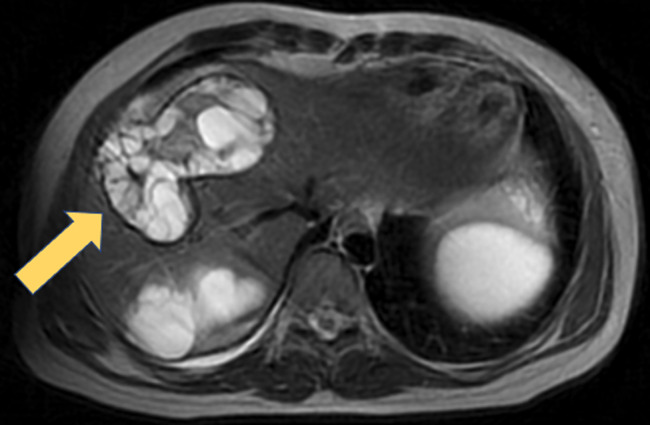
T2 sequence of magnetic resonance imaging of the upper abdomen; within the liver, there are multiple cysts, including several smaller cysts inside them (arrow); hepatomegaly is also noted; other cysts, containing smaller cysts were also noted in the peritoneal cavity between the intestinal loops, the parametrium and the ovaries

